# A Case of Small Bowel Obstruction Caused by Bezoars Diagnosed with Double Balloon Enteroscopy

**DOI:** 10.1155/2012/185489

**Published:** 2012-02-15

**Authors:** Masayuki Saita, Hiroshi Maekawa, Koichi Sato, Hajime Orita, Mutsumi Sakurada, Tomoaki Ito, Kunihiro Sinjoh, Yoshihiro Komatsu, Shigeo Nohara

**Affiliations:** Department of Surgery, Juntendo Shizuoka Hospital, Juntendo University School of Medicine, Nagaoka, Izunokuni-Shi, Shizuoka 410-2295, Japan

## Abstract

Primary small bowel bezoars are rare and cause acute abdomen due to small bowel obstruction (SBO). A 69-year-old Japanese man presented with epigastric pain associated with fullness. Physical examination of the abdomen showed no marked signs of peritoneal irritation. An erect X-ray film of the abdomen showed small bowel obstruction. Computed tomography (CT) showed a dilated small bowel loop proximal to the site of the obstruction. Retrograde double balloon enteroscopy (DBE) was performed and showed yellow, hard bezoars blocking the distal ileum. At surgery, a bezoar was found impacted in the distal ileum, and enterotomy with extraction was performed. After 9 days, the patient was discharged from our hospital in satisfactory condition. DBE also appears to be a safe and useful diagnostic tool in patients with SBO, and the findings of DBE influence the strategy of therapy in patients in whom the cause of SBO could not be determined by conventional radiography.

## 1. Introduction

Bezoars are an uncommon cause of small bowel obstruction (SBO) and are usually concretions of foreign material found in the stomach. They most often develop in patients who have undergone gastric surgery [1]. Bezoars usually become impacted in the narrowest potion of the small bowel, which is 50–75 cm proximal to the ileocecal valve, or at the valve itself [2]. Preoperative diagnosis of small bowel obstruction due to bezoars is difficult. Double balloon enteroscopy (DBE), a new insertion method developed in 2001, allows for complete visualization and therapeutic interventions for entire small bowel bezoars.

In this case, we could diagnose small bowel obstruction due to bezoars with DBE.

## 2. Case Report

**Figure 1:**
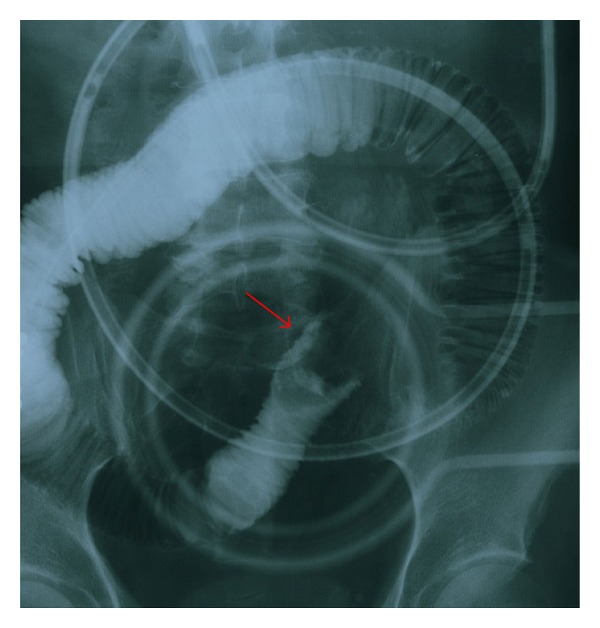
Contrast examination of the ileus tube shows total obstruction of the ileum by the mass.

**Figure 2:**
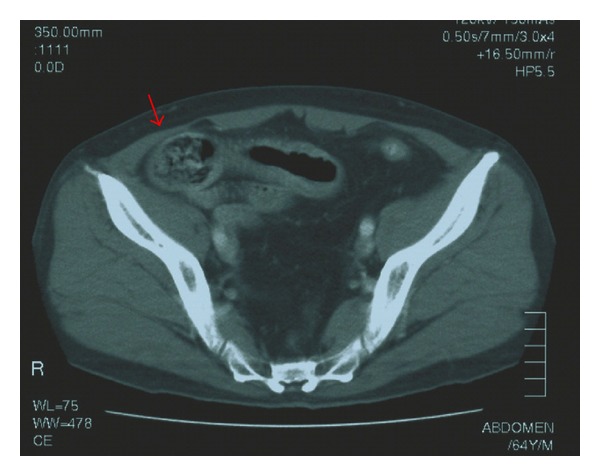
Pelvic CT shows a dilated small bowel loop and a mass measuring 4 cm.

**Figure 3:**
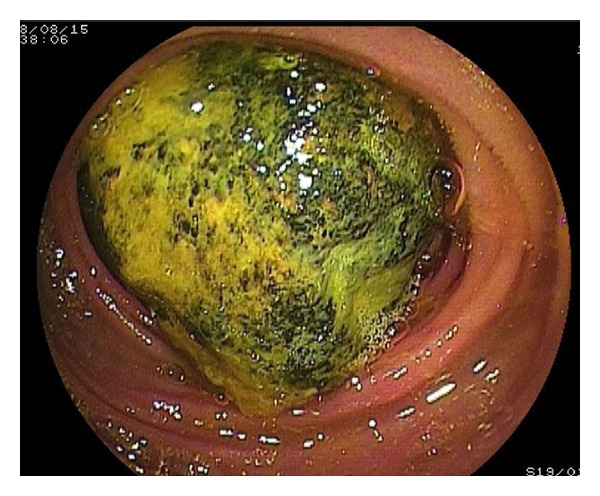
DBE shows yellow, hard bezoars blocking the distal ileum.

A 69-year-old Japanese man was admitted with epigastralgia. Physical examination of the abdomen showed marked generalized distention with diffuse tenderness but no signs of peritoneal irritation. The only exotic food he had eaten was persimmon. Laboratory data showed elevation of the white blood cell count to 9.200/mm^3^ and C-reactive protein of 8.0 mg/dL. Other chemistry and liver function tests were normal. An erect X-ray film of the abdomen showed small bowel gas. Contrast examination of the ileus tube showed total obstruction of the ileum with the mass (Figure 1). Computed tomography (CT) clarified a dilated small bowel loop and a mass measuring 4 cm (Figure 2). Seven days after the acute episode, retrograde DBE was performed and showed yellow, hard mass blocking the distal ileum (Figure 3); however, it was difficult to fragment the bezoars.

**Figure 4:**
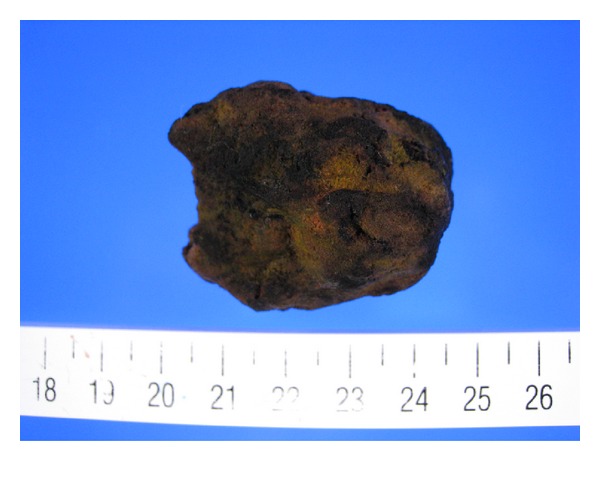
A bezoar impacted in the distal ileum.

 During the operation, a bezoar impacting the ileum, which is about 100 cm proximal to the ileocecal valve, was found, and extraction with enterotomy was performed. The bezoar was 4.0 × 3.0 × 3.0 cm, yellow and sclerous (Figure 4). Nine days after the operation, the patient was discharged from our hospital in satisfactory condition.

## 3. Discussion

Bezoars are usually found in the stomach, but they may also pass into the small bowel [3]. Small bowel bezoars are very rare and may cause acute abdomen due to obstruction [4]. The associated clinical signs and symptoms include vomiting, nausea, abdominal pain, fever, and elevated leukocyte count [5]. Bezoars are concretions of fruit and vegetable fiber in the alimentary tract [6]. Other predisposing factors are ingestion of high-fiber foods [7]. The patient's favorite food was persimmon. Overeating persimmons causes gastric bezoars [8]. Persimmon bezoars might therefore have caused small bowel obstruction.

SBO can be diagnosed by various modalities. To date, computed tomography (CT) imaging has been reported to be useful for imaging patients with SBO as this modality is very effective for determining the presence or absence of SBO as well as the level and cause of SBO [7]; however, there are only a few reports [9] regarding the CT findings of bezoars associated with SBO. DBE is a safe, feasible diagnostic tool that allows high-resolution endoscopic imaging and total enteroscopy [10] and enables the collection of tissue for histological studies. DBE also appears to be a safe and useful diagnostic tool in patients with SBO, and the findings of DBE influence the strategy of therapy in patients in whom the cause of SBO cannot be determined by conventional radiography [11]. Most bezoars in the stomach are treated by endoscopic destruction or removal. Endoscopic-guided electrohydraulic lithotripsy (EHL), which has gained acceptance in the treatment of biliary and urinary stones, was reported to be safe and effective for the treatment of large and hard gastric bezoars [12]. Recently, it was reported that DBE could recover entrapped endoscopy capsules in some patients [13]. In this case, we could diagnose SBO caused by bezoars with DBE before the operation. There are at least two references of the similar cases [10].

In conclusion, DBE is a feasible method for the management of bezoar-induced intestinal obstruction when performed by a well-trained and experienced surgeon.
